# Microbial Response to Fungal Infection in a Fungus-Growing Termite, *Odontotermes formosanus* (Shiraki)

**DOI:** 10.3389/fmicb.2021.723508

**Published:** 2021-11-22

**Authors:** Chen-Yu Wu, Jing Meng, Austin Merchant, Yi-Xiang Zhang, Mu-Wang Li, Xu-Guo Zhou, Qian Wang

**Affiliations:** ^1^Shanghai First Maternity and Infant Hospital, Tongji University School of Medicine, Shanghai, China; ^2^School of Biotechnology, Jiangsu University of Science and Technology, Zhenjiang, China; ^3^Department of Entomology, University of Kentucky, Lexington, KY, United States; ^4^CAS Key Laboratory of Insect Developmental and Evolutionary Biology, CAS Center for Excellence in Molecular Plant Sciences, Chinese Academy of Sciences, Shanghai, China; ^5^CAS Center for Excellence in Biotic Interactions, University of Chinese Academy of Sciences, Beijing, China

**Keywords:** *Odontotermes formosanus*, *Metarhizium robertsii*, 16S rDNA sequencing, metagenome, gut microbiota

## Abstract

The crosstalk between gut microbiota and host immunity has emerged as one of the research foci of microbiome studies in recent years. The purpose of this study was to determine how gut microbes respond to fungal infection in termites, given their reliance on gut symbionts for food intake as well as maintaining host health. Here, we used *Metarhizium robertsii*, an entomopathogenic fungus, to infect *Odontotermes formosanus*, a fungus-growing termite in the family Termitidae, and documented changes in host gut microbiota *via* a combination of bacterial 16S rDNA sequencing, metagenomic shotgun sequencing, and transmission electron microscopy. Our analyses found that when challenged with *Metarhizium*, the termite gut showed reduced microbial diversity within the first 12 h of fungal infection and then recovered and even surpassed pre-infection flora levels. These combined results shed light on the role of gut flora in maintaining homeostasis and immune homeostasis in the host, and the impact of gut flora dysbiosis on host susceptibility to infection.

## Introduction

*Metarhizium* is an entomopathogenic fungus widely present in the natural environment, whose conidia come into contact with the surface of the insect cuticle through the secreted protein MAD1 adhesin (Wang and St Leger, 2007). Afterward, several enzymes such as chitinase are secreted and mechanical pressure is applied causing the attached fungal cell to produce hyphae that penetrate the cuticle and shed budding spores from the hyphae into the body of the host. Colonization begins when conidia reach the hemolymph (Schrank and Vainstein, 2010) and involves the production of hydrolytic enzymes that assimilate nutrients and toxins with immunosuppressive activity (Gillespie et al., 2000; Freimoser et al., 2003; Pal et al., 2007). If the insect is unable to remove the conidia from its body in time, *Metarhizium* will invade and interact with the host immune system, interfering with, disrupting or manipulating its composition or inducing immune defenses (Lu and St Leger, 2016). Meanwhile, conidia multiply in the body cavity and produce secondary metabolites to damage the host (Vivekanandhan et al., 2020), which can lead to host death.

When the fungus successfully invades the insect cuticle, it causes activation of the insect’s immune system, which includes humoral responses (e.g., production of antimicrobial peptides) and cellular responses (e.g., encapsulation and melanization). Innate defenses cannot respond to environmental changes and do not have adaptive characteristics (Lemaitre and Hoffmann, 2007; Kounatidis and Ligoxygakis, 2012; Lu and St Leger, 2016). In *Drosophila melanogaster*, gene mutations modifying disease resistance principally affect fast-acting, generalized immune responses such as coagulation, phagocytosis, encapsulation, and melanization rather than specific, slow-acting responses such as antifungal peptide production (Lu et al., 2015). Insects show active avoidance of pathogenic fungi before they break through the cuticle barrier, which is particularly evident in group-living social insects. In these species, social immunity occurs at the group level, which is manifested by positive social contact between individuals grooming each other, corpse removal, and use of oral gland secretions. This preventive behavior may reduce the selective pressure to increase the number of immune genes in the genomes of social insects (Smith et al., 2011a,b). However, social and innate immunity are not independent of each other (Liu et al., 2015). Studies have confirmed that social immunity depends in part on the regulation of exogenous enzymes associated with the body’s immune system (Esparza-Mora et al., 2020).

Symbiotic gut microbes are present in a wide variety of insect taxa and provide benefits to their hosts ranging from enhanced digestion to accelerated developmental time (Engel and Moran, 2013). In addition, gut microbes can promote the establishment of innate immunity in the body and are critical to maintaining the homeostasis of the gut immune system (Lee, 2008; Douglas, 2018). Contributions of gut microbiota to immunity include mediation of the immune response, increased immune tolerance, and maintenance of immunostasis to infectious diseases (Charroux and Royet, 2012; Erkosar and Leulier, 2014). Current studies have identified symbiotic fungi in the guts of termites that have antimicrobial activity and can inhibit infections targeting their hosts (Sapountzis et al., 2015; Xu et al., 2020). Peterson sequenced the gut contents of the lower termite *Reticulitermes flavipes* after infestation with the entomopathogenic fungus *Beauveria bassiana* and found that the termite gut produces an oxidative response to reactive oxygen species (West et al., 2011; Buchon et al., 2013; Peterson and Scharf, 2016b) and that enzyme secretion by gut flora is involved in gut oxidative processes (Imlay, 2013), which may be a mechanism for endogenous antifungal defense in termites. In particular, temporal expression of symbiont-derived amidohydrolases may contribute to resistance and immunity to fungal infection (Peterson and Scharf, 2016b).

The overall goal of this study was to determine how gut microbiota respond to fungal infection in termites, given their reliance on gut symbionts for food intake as well as maintaining host health. Here, we used *Metarhizium robertsii*, an entomopathogenic fungus, to infect *Odontotermes formosanus*, a fungus-growing termite in the family Termitidae. Building on the literature and preliminary research, we hypothesized that the gut flora would experience destabilization for a certain period following host infection, eventually returning to equilibrium as a result of gut symbiont intervention. To test this hypothesis, we integrated conventional pathological observation using transmission electron microscopy (TEM) with advanced diagnostic-omics toolsets, including 16S rDNA sequencing and metagenomic shotgun sequencing.

## Materials and Methods

### Sampling

*Odontotermes formosanus* colonies were collected from rotting wood in Wuhan, China, in August 2019. To provide food and shelter, *O. formosanus* field colonies were provisioned with filter paper and acclimated for 1-day to filter out low-activity individuals before they were used in the subsequent experiments. Changing the diet from their natural food (e.g., wood logs) to filter paper could potentially influence *O. formosanus* gut microbiome, and therefore, the acclimation time was within 24 h. *M. robertsii* ARSEF2575 was obtained from the Institute of Plant Physiology and Ecology, Chinese Academy of Sciences, Shanghai. *M. robertsii* spores, which were grown on PDA culture media, were washed with 0.1% Tween 80 solution and sample concentration was measured with a hemocytometer, then stored at 4°C as a spore suspension. The spore suspension was diluted to the desired concentration (108 spores/ml) and sprayed on a piece of filter paper, which was placed inside a Petri dish with 12 cm diameter. Termites were allowed to crawl on the filter paper to infect them and were constantly exposed to *M. robertsii* spores for the duration of the experiment. The control group termites were given a filter paper sprayed with 0.1% Tween 80 solution and otherwise kept in the same conditions as the treatment group. For the survival experiment, 40 worker termites were used per group in each of 3 replicates from different termite colonies. Termites were counted every 6 h until all treatment group termites had died.

Sampling was conducted in parallel with the survival experiment. Termites exposed to *M. robertsii* spores were dissected at 0, 12, 24, 48, and 72 h post-infection. We placed termites on ice to anesthetize them, then rinsed them with sterile water for 1 min, disinfected them with a 70% alcohol rinse twice for 1 min each time, then washed them once for 1 min again with sterile water. A drop of phosphate-buffered saline (PBS) was placed at the center of the dissection table. After dissecting the hindgut under aseptic conditions, the gut tissue was gently torn with dissecting forceps and rinsed several times to expose the contents to PBS and achieve suspension of the gut contents.

### Transmission Electron Microscopy Analysis

Hindgut samples were fixed and preserved in fixative for TEM. Samples were sequentially dehydrated in 30, 50, 70, 80, 95, and 100% alcohol for 20 min in each solution; then immersed in 100% acetone twice, each time for 15 min. Sections were cut into resin blocks after infiltration embedding and polymerization. Sections were immersed in 2% uranium acetate saturated alcohol solution in darkness for 8 min, rinsed in 70% ethanol three times, and then rinsed in ultrapure water three times. Sections were then immersed in 2.6% lead citrate for 8 min to avoid CO_2_ staining, and then rinsed with ultrapure water three times. After being dried with filter paper, the sections were placed in a grid board and dried overnight at room temperature. The sections were observed under a transmission electron microscope and images were collected for analysis.

### Total DNA Extraction From Samples and Quantification of Microbial DNA

Termite gut contents were collected into Cell and Tissue Lysis Buffer (Ambion, Austin, TX, United States) and homogenized with a pestle before being frozen at −80°C. DNA was extracted using the Qiagen (Germantown, MD, United States) QIAamp 96 DNA QIAcube HT Kit with the following modifications: enzymatic digestion with 50 μg of lysozyme (Sigma, St. Louis, MO, United States) and 5 U each of lysostaphin and mutanolysin (Sigma) for 30 min at 37°C followed by beadbeating with 50 μg of 0.1 mm diameter zirconium beads for 6 min on a TissueLyzer II (Qiagen) prior to loading onto the QIAcube HT Kit. DNA concentration was measured using the Qubit dsDNA HS Assay Kit (Invitrogen, Carlsbad, CA, United States).

### 16S rRNA Sequencing

The V4 region of the 16S rRNA gene was amplified using primers 515F (5′-GTGCCAGCMGCCGCGG-3′) and 806R (5′-GGACTACHVGGGTWTCTAAT-3′) that were tailed with Illumina adapter sequences and index tags to facilitate sample pooling (Sato et al., 2017). Library quality was assessed on a Qubit 2.0 Fluorometer (Thermo Scientific) and Agilent Bioanalyzer 2100 system. The library was then sequenced on an Illumina NovaSeq platform and 250 bp paired-end reads were generated.

### Metagenomic Shotgun Sequencing

Illumina libraries were created using a Nextera XT DNA Library Prep Kit (Illumina, San Diego, CA, United States) with reduced reaction volumes: 200 pg of DNA were used (160 pg/μL × 1.25 μL), and tagmentation and PCR reagent volumes were reduced to 1/4 of the standard volumes. Tagmentation and PCR reactions were carried out according to the manufacturer’s instructions. The reaction mixtures were then adjusted to 50 μL by adding dH_2_O, and AMPure (Beckman Coulter) Cleanup was carried out as per the manufacturer’s instructions. Libraries were then sequenced with 2 × 150 bp paired end reads on an Illumina HiSeq 2500.

Sequencing adapters and low-quality bases were removed from the sequencing reads using scythe (v0.994) (Manousaki et al., 2019) and sickle (v1.33) (Acharya et al., 2016), respectively, with default parameters. Host reads were then filtered by mapping all sequencing reads to the termite reference genome using bowtie2 (v2.2.8) (Jaiswar et al., 2018) under “very-sensitive” mode. Unmapped reads were used for downstream analyses. Characteristics of the shotgun metagenomic sequencing data are summarized in Supplementary Table 1.

### 16S rDNA Gene Amplicon Sequence Analysis

De-multiplexed and quality trimmed 16S rRNA gene amplicon reads obtained from a NovaSeq sequencer were processed with MacQIIME (v1.9.1) (Shoskes et al., 2016). The reads were clustered into operational taxonomic units (OTUs) at a 97% similarity cutoff with the pick_open_reference_otus.py function provided by USEARCH (v6.1.554) (Edgar, 2010), using the SILVA 123 database (Thijs et al., 2017) release as a reference. The OTUs were filtered with the filter_otus_from_otu_table.py function, resulting in a total of 1,938 OTUs for all 15 samples (3 replicates each for the control and 12, 24, 48, and 72 h post-infection groups). The taxonomic composition of the samples was visualized with the Phyloseq package for R (v4.0.0^1^) (McMurdie and Holmes, 2013). The beta diversity metrics of the 15 samples were calculated using the beta_diversity function of unweighted UniFrac provided by QIIME2 (Hall and Beiko, 2018; Mohsen et al., 2019). Sample dissimilarity matrices were visualized on principal coordinate analysis (PCoA) plots through Phyloseq and clustered heat maps were visualized using the clustermap function in Seaborn (v0.8) (Mohsen et al., 2019) (method = “average”, metric = “correlation”). Group significance was determined with the PERMANOVA function available through the vegan package for R (v4.0.0). Relative similarity between metadata categories (harvest dates) was calculated with Phyloseq, which summarized the distances between pairs of sample groups (from weighted or unweighted UniFrac dissimilarity matrices), and then a two-sided Student’s two-sample *t*-test was performed to evaluate the significance of differences between the distances. Relative abundances of phyla and domain taxa were computed from the sum of abundances of OTUs within their respective taxonomies, and group significance was calculated with a two-sided Student’s two-sample *t*-test. Detailed scripts for the entire analysis pipeline can be found at https://github.com/joey711/phyloseq.

### Functional Annotation

Metagenomic sequences were annotated to functional categories against the Kyoto Encyclopedia of Genes and Genomes (KEGG^2^) database using BLAST, and results were selected based on their BLAST Coverage Ratio (BCR) (Kanehisa et al., 2016, 2017). The BCR of reference and query gene was calculated with cutoff at ≥40%. BCR was calculated as:

$$\begin{array}{c} \text{BCR}\left(\text{Ref}.\right)=\left(\text{Match}/\text{Length}\left(\text{R}\right)\right)\times 100\%;\\ \text{BCR}\left(\text{Que}.\right)=\left(\text{Match}/\text{Length}\left(\text{Q}\right)\right)\times 100\%, \end{array}$$

where Match is the available alignment length between reference and query genes, Length(R) is the length of the reference gene, and Length(Q) is the length of the query gene. Data from the annotations were imported into the Statistical Analysis of Metagenomic Profiles (STAMP) (version 2.1.3) package for statistical analysis and visualization. Differences were considered significant at *P* < 0.05 using a *t*-test (Kanehisa, 1997; Kanehisa et al., 2004, 2006).

### Statistical Analysis

The following statistical analyses were performed for both 16S rRNA gene and shotgun metagenomic sequencing data. Detection of differentially abundant bacterial phyla between methods was performed using the Phyloseq and DESeq2 packages for R (v4.0.0) (Love et al., 2014). PERMANOVA (adonis) tests [*via* the vegan package for R (v4.0.0)] were also performed using Bray–Curtis distances to test for a potential association between sampling methods and microbiota composition. For all statistical analyses, *P*-values < 0.05 were considered significant.

For 16S rRNA gene sequencing data, a filtered OTU count table was created using R (v4.0.0). Normalized [x/(sum (x)] OTU values from the same table were used for the PERMANOVA test.

For shotgun metagenomic sequencing data, values from the created count table [based on the estimated number of reads from bacterial genera calculated by MetaPhlAn (v2.038)] were used for DESeq2 analysis (Varet et al., 2016). For the PERMANOVA test, proportional abundances [calculated by MetaPhlAn (v2.038)] were used. Gene richness differences (shotgun metagenomic sequencing data only) were assessed using the Wilcoxon signed-rank test.

The following statistical analyses were performed for shotgun metagenomic sequencing data only. Using MicrobiomeAnalyst, the Shotgun Data Profiling (SDP) module offers a similar set of methods for pattern discovery and comparative analysis of gene abundance data produced from either predictive functional profiling or metagenomics (Supplementary Table 2). A unique feature of the SDP is its functional annotations based on modules, pathways, and metabolic networks. MicrobiomeAnalyst enables users to easily visualize the distribution of these functions across samples and study conditions. It also supports explicit statistical testing to identify enriched functions (Goeman et al., 2004). Users can interactively explore the results within a metabolic network environment for further functional insights (Kanehisa et al., 2012).

## Results

### Disruption of Termite Gut Flora Caused by *Metarhizium robertsii* Infection

**FIGURE 1:**
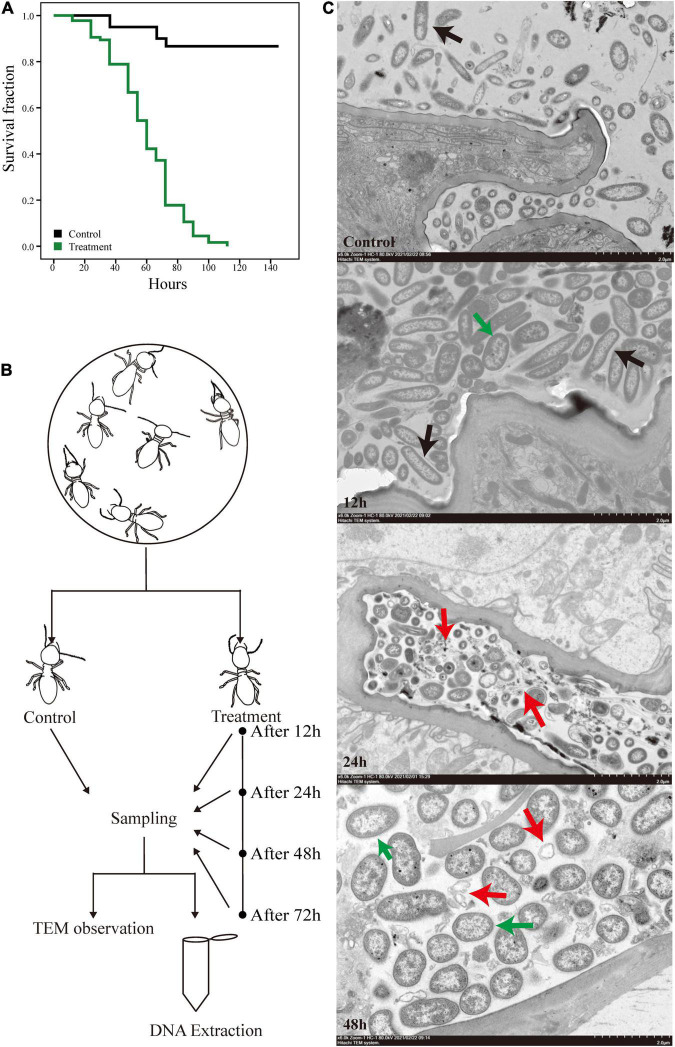
Experimental design and the survival curves of *O. formosanus* during *M. robertsii* infection. **(A)** Kaplan–Meier survival curve of fungus treatment and control groups. **(B)** Experimental design. **(C)** Transmission electron microscopy (TEM) images of the hindguts of individuals from treatment groups after 12, 24, and 48 h of *M. robertsii* infection, in addition to those of individuals from uninfected control groups. All images were captured from a HITACHI transmission electron microscope (Model HT7800; scale bar: 2 μm). Black arrows: gut microorganisms in the form of long strips or pikes; red arrows: microorganism lysis debris; green arrows: *M. robertsii* spores.

We exposed *O. formosanus* termites to pathogenic *M. robertsii* spores and observed the effects of infection on the host and its gut flora. The survival rate of termite hosts post-infection is shown in Figure 1A. The termite population began to show mortality at 12 h post-infection, which reached approximately 10–20% at 24 h, 30–40% at 48 h, and over 80% at 72 h. Mortality in uninfected control groups fed 0.1% Tween 80 solution as a water source remained below 20% for the duration of the experiment. Following measurement of post-infection survivorship, the hindguts of infected termites were dissected and collected at 12, 24, 48, and 72 h post-infection alongside those of uninfected controls. The hindguts of these five groups were then observed under an electron microscope. At the same time, an additional 100 hindguts were dissected from each group for mixing and subjected to 16S and metagenome sequencing. The whole experimental procedure is shown in Figure 1B.

The electron microscope observations provide a glimpse of the changes in gut tissues and microorganisms following *M. robertsii* infection. Compared with the control group, a higher density of microorganisms was observed at 12 h post-infection, with a proliferation of microorganisms in the form of long strips or pikes (Figure 1C, black arrows). Twenty-four hours after the infection of the host by *M. robertsii*, some microorganisms underwent lysis and their edges became distorted and irregular (Figure 1C, red arrows), implying that some of the microbes did not adapt to the invasion of *M. robertsii* and died. At 48 h post-infection, there was a large amount of bacterial lysis debris present in the gut (Figure 1C, red arrows), and *M. robertsii* occupied a large proportion of the remaining space, squeezing the survival space of other gut microorganisms.

Overall, based on our observation, termites suddenly died at specific time points, reflected by stepwise mortality during *M. robertsii* infection. The presence of spores in the gut indicated that *M. robertsii* can invade the gut to cause damage to the host over a period. However, *M. robertsii* spores did not break through the gut, and no significant damage was observed in the gut tissues.

### 16S rDNA Sequencing and Metagenomic Overview of Termite Gut Microbiota

To study the changes in the gut microbial community of termites infected by *M. robertsii*, we performed 16S rRNA and metagenomic shotgun sequencing. The V4 region was selected as the target region for PCR amplification, followed by sequencing analysis and strain identification of the hypervariable region. Through splicing reads, an average of 87,721 tags were measured per sample. This number was reduced through quality control to an average of 85,053 valid tags per sample resulting in an effective quality control data volume of 74,691 and quality control efficiency of 85.05%. Sequences were clustered into a total of 1,938 OTUs with 97% identity. The number of OTUs able to annotate to the SILVA 123 database was 1,865 (96.23%). The proportion of OTUs annotated to the kingdom level was 96.23%, while the proportion annotated to the phylum level was 86.12%. Annotation results under the remaining biological classification levels are shown in Table 1.

**TABLE 1:** Results of OTU annotation under different biological classifications.

Annotation type	Annotation proportion (%)
Database	96.23
Kingdom level	96.23
Phylum level	86.12
Class level	82.66

The Illumina HiSeq platform was used for metagenomic sequencing. The total sequencing data volume was 111,464.26 Mbp and the average sequencing data volume was 7,430.95 Mbp.

The effective quality control percent was 99.87%; see Table 2 for the remaining gene assembly information. For each sample and mixed assembly, we used MetaGeneMark software to carry out gene prediction. Gene prediction yielded 3,742,816 open reading frames (ORFs) of average length 233,926 Mbp. This number was reduced to 792,038 ORFs with a total length of 472.72 Mbp after redundancy, of which the number of complete genes was 214,957, accounting for 27.14% of ORFs after redundancy. Non-redundant gene sets were compared against the MicroNR library for blastp and species annotation using an LCA algorithm, and the ratio of annotation to genus and phylum was 51.81 and 74.77%, respectively.

**TABLE 2:** Metagenomic sequencing results.

Data type	Data size
Total raw data	111,464.26 Mbp
Total clean data	111,320.04 Mbp
Scaffolds (average)	168,871
Total scaffolds length (nt)	3,039,383,189 bp
N50 scaffolds length (nt)	1,191.31 bp
N90 scaffolds length (nt)	574.50 bp
Scaftigs (average)	141,985
Total scaftigs length (nt)	2,450,635,224 bp
N50 scaftigs length (nt)	1,110 bp
N90 scaftigs length (nt)	565 bp
Total ORFs	3,742,816
Number of complete ORFs	214,957
Total raw data	111,464.26 Mbp
Total clean data	111,320.04 Mbp
Scaffolds (average)	168,871
Total scaffolds length (nt)	3,039,383,189 bp
N50 scaffolds length (nt)	1,191.31 bp
N90 scaffolds length (nt)	574.50 bp
Scaftigs (average)	141,985
Total scaftigs length (nt)	2,450,635,224 bp
N50 scaftigs length (nt)	1,110 bp
N90 scaftigs length (nt)	565 bp

### Changes in Flora Diversity Caused by *Metarhizium robertsii* Infection

**FIGURE 2:**
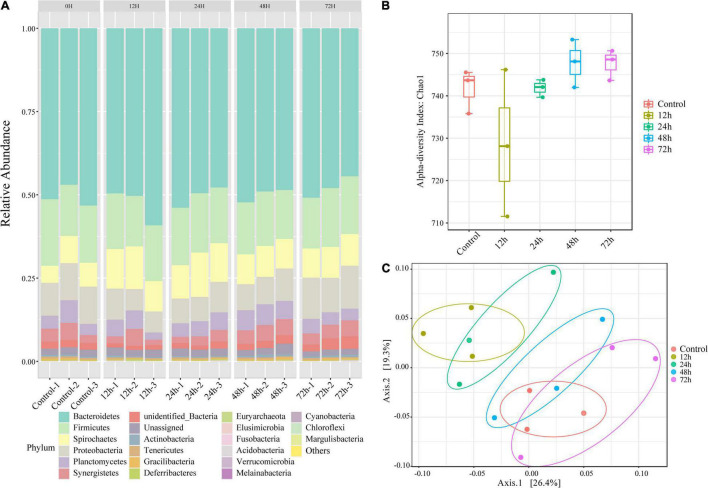
Changes in the composition of *O. formosanus* gut microbiome following *M. robertsii* infection based on 16S rRNA sequencing data. **(A)** Relative abundances of gut microbes at the phylum level. There are five clusters, each including three independent repetitions. Each bar graph demonstrates the microbial community composition of one independent repetition in the corresponding group. **(B)** Alpha-diversity comparison based on the Chao1 diversity index. **(C)** Principal coordinate analysis of gut microbial community composition was based on unweighted UniFrac distance. Three replications of the same treatment group were represented by dots of the same color. There were four treatment groups and one control group.

Using the results of our 16S rRNA sequencing analysis, we investigated the changes in gut microorganism community composition in termites exposed to *M. robertsii* spores. Despite variations in community composition, six phyla comprised the majority of termite gut microbiota at all time points (mean abundance over 1,000) (Figure 2A). In descending order by abundance, these phyla were Bacteroidetes, Firmicutes, Proteobacteria, Spirochaetes, Planctomycetes, and Synergistetes. The status of the most dominant phylum, Bacteroidetes, did not change during *M. robertsii* infection. At 12 h post-infection, Spirochaetes outnumbered Proteobacteria. At 48–72 h post-infection, Proteobacteria regained its dominance over Spirochaetes, while increases in the abundance of these two phyla squeezed the survival space of Planctomycetes. The calculated non-parametric alpha diversity index (Chao1) further confirms the observed trend (Figure 2B). A significant reduction in the diversity of gut flora was observed at 12 h post-infection, followed by a slow recovery to pre-infection diversity levels.

To increase the reliability of the results, we tested for within-group differences. PCoA (Figure 2C) shows bacterial community clusters for each treatment group and the control group. Among these five groups, the 12 h group can be separated to form independent clusters. The clusters of the 12 and 24 h phylum composition samples partially overlap, distinguished from the remaining three groups that overlap each other. Calculation of unweighted UniFrac distances revealed significant differences between the control and treatment groups after 72 h of *M. robertsii* infection, and between the treatment groups after 12 and 72 h of *M. robertsii* infection (Supplementary Figure 1).

We also looked at the compositional changes in *O. formosanus* gut microbial community following *M. robertsii* infection based on the metagenome data (Supplementary Figure 2). The resultant trends at different time points were generally consistent with the 16S rRNA sequencing data, especially for the phyla of Acidobacteria, Actinobacteria, Deferribacteres, Elusimicrobia, Firmicutes, Fusobacteria, Planctomycetes, and Proteobacteria. Gracilibacteria was not detected in the metagenome data, possibly due to the differences in library preparation between the two sequencing approaches.

### Trends Over Time in Significantly Changed Phyla After *Metarhizium robertsii* Infection

**FIGURE 3:**
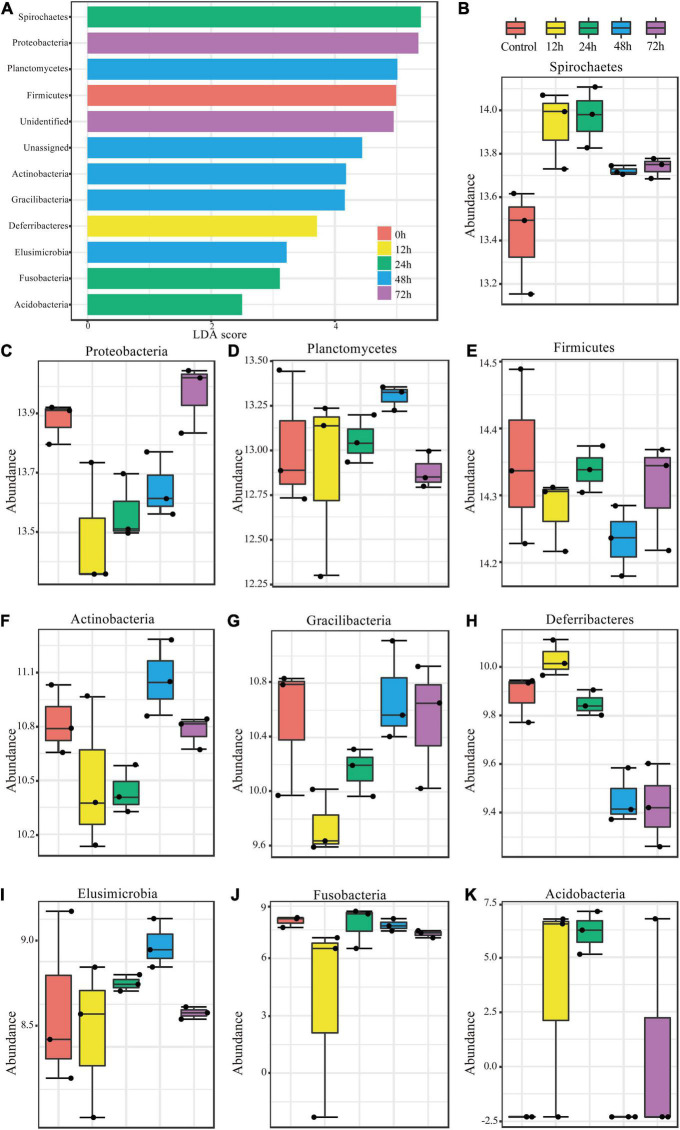
Significant changes in phylum abundances and their specific trends over time. **(A)** Linear discriminant analysis (LDA) combined with effect size measurements (LEfSe) was used to screen qualified OTUs. They showed statistically different strains with LDA scores greater than a prespecified value of 2. Different colors refer to different time periods. **(B–K)** Relative abundances over time of the significantly different phyla.

To investigate the changes in abundance within termite gut microbe phyla following *M. robertsii* infection, we measured the linear discriminant analysis (LDA) effect size (LEfSe), the statistical significance through coupled standard tests, and performed additional analyses to test for biological consistency and effect correlation. The LDA score was used to identify phyla exhibiting the greatest amount of variation in abundance levels during the experimental period. An LDA score greater than 2 was used as an indicator for high variation. High variation phyla were, from highest to lowest LDA score, Spirochaetes, Proteobacteria, Planctomycetes, Firmicutes, Actinobacteria, Gracilibacteria, Deferribacteres, Elusimicrobia, Fusobacteria, and Acidobacteria (Figure 3A). Bacteroidetes, the most abundant phylum found through our analysis (Figure 2A), showed minimal variation in abundance post-infection.

The variations in abundance within individual phyla during the experimental period are shown in Figures 3B–K. Spirochaetes, Deferribacteres, and Acidobacteria showed similar trends (Figures 3B,H,K), all of which increased in abundance to a peak at 12 h post-infection and then decreased. Proteobacteria and Gracilibacteria showed the opposite trend (Figures 3C,G), in which abundance dropped to its minimum at 12 h and then increased thereafter. Planctomycetes (Figure 3D) abundance changed in a fluctuating pattern opposite to that observed in Firmicutes (Figure 3E). Abundance trends in other phyla are shown in Figures 3F,I,J.

### The Interactions Among Phyla During *Metarhizium robertsii* Infection

**FIGURE 4:**
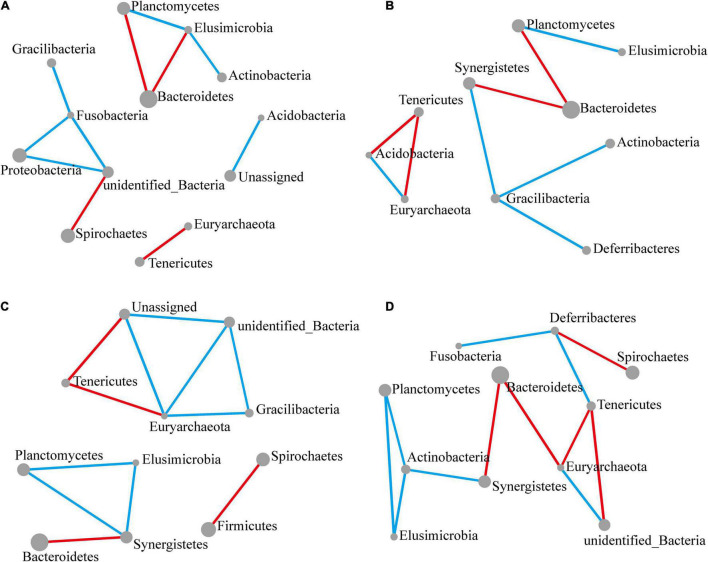
Significant changes in interactions at the phylum level. The size of each node reflects the abundance of each phylum, while the width of each line connecting two nodes corresponds to the strength of the interaction between the phyla representing each node. A blue line indicates that interactions were positive, while a red line indicates that interactions were negative. In all cases, the control group was used as a comparison and Pearson’s product-moment correlation was used to determine interaction strength (*P*-value ≤ 0.05). **(A–D)** Network diagram showing interactions at the phylum level, respectively, at 12 **(A)**, 24 **(B)**, 48 **(C)**, and 72 h **(D)** following *M. robertsii* infection.

In the previous section, we observed similar and opposing trends in abundance over time between phyla, implying positive and negative interactions between these groups in the context of *M. robertsii* infestation. To further compare interactions between phyla before and after infestation, we used the metagenome sequencing results to construct correlation network diagrams. We compared phylum abundance profiles from control groups with those from other time periods and constructed correlation network diagrams to visualize and compare microbial communities among phyla. Using Pearson’s product-moment correlation, significantly related phyla were screened out for individual analysis (*P*-value < 0.05) and network diagrams were produced (Figure 4).

After 12 h of *M. robertsii* infestation, Planctomycetes, Elusimicrobia, Bacteroidetes, and Actinobacteria abundances showed significant interaction. At the same time point, Gracilibacteria, Fusobacteria, Proteobacteria, and Spirochaetes abundances showed significant interaction. Overall, positive correlations occurred more intensively than negative correlations (Figure 4A). After 24 h of *M. robertsii* infestation, the interaction between Gracilibacteria and Fusobacteria was destroyed, while Gracilibacteria, Actinobacteria, and Deferribacteres formed part of a new correlation network (Figure 4B). The destruction and establishment of other interactions is shown in Figures 4C,D. After 72 h of *M. robertsii* infestation, the interactions of the phyla tended to form a large complex whole consisting of mostly positive correlations (Figure 4D). Five phyla (Planctomycetes, Elusimicrobia, Tenericutes, Euryarchaeota, and Bacteroidetes) showed consistent interaction. Among these phyla, Planctomycetes, and Elusimicrobia abundances were positively correlated, while Tenericutes and Euryarchaeota abundances were negatively correlated. In this study, the interactions among phyla were inferred from bioinformatics analyses. Direct evidence, however, will come from empirical ecological tests in the future.

### Infection of *Metarhizium robertsii* Leads to Changes in the Function of the Gut Flora

**FIGURE 5:**
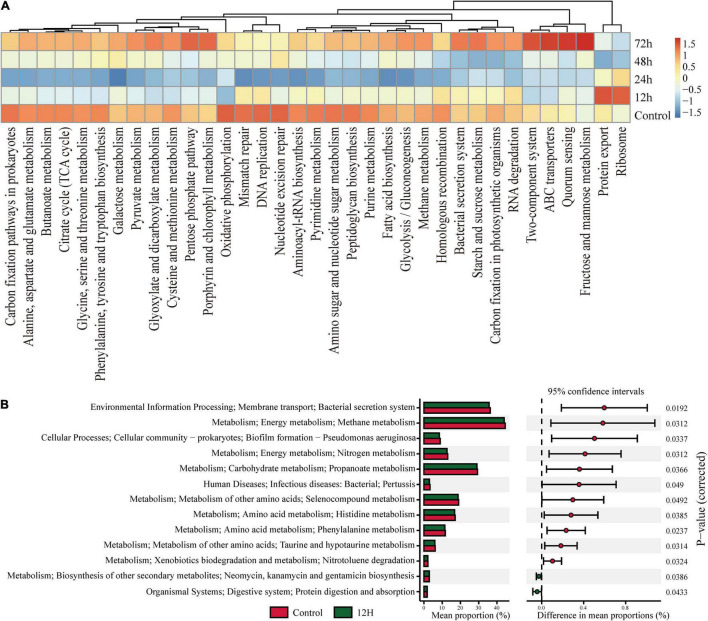
KEGG Orthology (KO) pathway enrichment among experimental groups. *Q*-value is used as a corrected *P*-value ranging from 0.0 to 1.0. Low *Q*-values indicate greater intensity (enrichment). **(A)** Heat map showed abundances of KO labels. The results were picked with minimum value of the BCR. **(B)** Prediction of KEGG pathways between control and treatment groups as detected by STAMP software. Differential KEGG pathways between the control group and the treatment group after infection at 12 h. For each comparison, the mean proportion of predicted KEGG pathways (left) and difference in mean proportions (right) were illustrated.

To investigate the functional changes in termite gut communities instigated by *M. robertsii* infection, we analyzed the functional potential of termite gut flora using our metagenomic data in concert with the KEGG Orthology (KO) Database. Figure 5 describes the pathways annotated using existing KO Database information on termite gut microbes. Cluster analysis showed that a total of 35 pathways (Figure 5A) exhibited an increase or decrease in functional genes at different time periods. Most metabolic functional genes decreased at 12 h post-infection and returned to pre-infection levels at 72 h post-infection. Some metabolic pathways such as Porphyrin and chlorophyll metabolism, Pentose phosphate pathway, Fructose and mannose metabolism, Quorum sensing, ABC transporters, Two-Protein export, and Ribosome in functional genes were significantly increased at 12 h post-infection. We found a decrease of the abundance and diversity of the gut flora 12 h after *M. robertsii* infection, so we additionally compared this treatment group with the control. Specifically, pathways labeled as Kanamycin and gentamicin biosynthesis and protein digestion and absorption were found to be highly enriched at 12 h post-infection, while those labeled as bacterial secretion system, Methane metabolism, Biofilm formation – *Pseudomonas aeruginosa*, Nitrogen metabolism, Propanoate metabolism, Human Diseases, Selenocompound metabolism, Histidine metabolism, Phenylalanine metabolism, Taurine and hypotaurine metabolism, and Nitrotoluene degradation were significantly inhibited (Figure 5B).

## Discussion

The aim of this study was to investigate how termite gut microbe populations change in response to pathogenic infection. We found that at 12 h post-infection the termites started to die, with approximately 80% mortality observed by 72 h post-infection. We observed changes in the gut colonies of the termites during *M. robertsii* infestation *via* TEM (Figure 1C). Morphologically, we did not observe the production of *M. robertsii* mycelia, suggesting that *M. robertsii* did not multiply in the hindgut (Wang and St Leger, 2007; Schrank and Vainstein, 2010). During the infection process, the gut cells and gut epithelial barrier remained largely intact, suggesting that *M. robertsii* entered the gut through termite feeding but did not cause damage to the gut epithelial cells. The intactness of the termite epithelial barrier was unexpected. In existing studies, disturbances in the gut flora caused by external conditions have been found to lead to disruption of the epithelial barrier (antibiotic treatment, dietary treatment in mammals, etc.) (Dillon et al., 2010; Tawidian et al., 2019). However, *Lutzomyia longipalpis*, a sandfly species within the family of Psychodidae, de-infected with *Metarhizium* was found to survive without disruption of the gut barrier (Sant’Anna et al., 2014). Throughout the infection of termites by *M. robertsii* (i.e., after the death of most individuals in the colony), no disruption of the epithelial barrier was observed in our electron microscope observations, suggesting that the gut environment and the presence of gut microorganisms are not conducive to the survival of *M. robertsii*, and that infestation of the host from the body surface into the hemolymph remains the main mode of infestation.

The mechanism by which *Metarhizium* infects termites from the body surface has been elucidated (Syazwan et al., 2021). In the natural environment, termites remove pathogenic fungi when they encounter them through social behaviors such as grooming, and this behavior also leads to the entry of pathogens into the digestive tract. It has been documented that fungal hyphae have difficulty penetrating the gut lumen (Chouvenc et al., 2009; Syazwan et al., 2021), that the hindgut lumen is the last structure invaded by *Metarhizium* mycelium after termite death, and that fungal disease is promoted in the carcass, which provides theoretical support for the difficulty of colonization of the gut by *Metarhizium*. A plausible reason may be the presence of β-1,3 glucanase (β-1,3 glus), an enzyme that breaks down β-1,3 glucan, a major component of the fungal cell wall (Brown and Gordon, 2005). Both termite-derived and symbiotic sources of β-1,3 glus may have fungal activity, thereby reducing the susceptibility of termite hosts to fungal diseases (Bulmer et al., 2009, 2012; Rosengaus et al., 2014). The response of the gut to resistance by *Metarhizium* is not homogeneous. The cuticle on the surface of the foregut has the potential to bind to *Metarhizium* conidia (Bulmer et al., 2009), and termicin, an antibacterial substance secreted by the salivary glands, causes significant inhibition of *Metarhizium* activity. Termicin is most effective against filamentous fungi (Da et al., 2010). Its expression in the salivary glands suggests that while this inhibitory activity in the foregut may arise from upstream glandular secretions, the gut environment is not conducive to spore germination. The midgut has the highest protease activity and amino acid content (Fujita and Abe, 2010), and measures by the anterior midgut greatly reduce the damage to the gut caused by *Metarhizium*. This specific structure of the gut can prevent *Metarhizium* from attacking the host through the interior of the gut. The hindgut has a much larger number of microorganisms than the anterior midgut, so the antimicrobial activity of the hindgut can be largely attributed to its endosymbionts, as they occur only in the hindgut. The physiological environment of the gut is also correlated with microbial abundance. In the hindgut, oxide levels are inversely related to microbiota abundance (Sousa et al., 2019), which could also indicate that an anaerobic hindgut environment created by high hindgut microbiota abundance is not conducive to the growth of *Metarhizium*. Moreover, the hindgut is located close to the rectum and the feces are also antimicrobially active, although the source of this antimicrobial activity is not known. The growth of *Metarhizium* can also be reduced in feces mixed with complex microorganisms *in vitro* (Rosengaus et al., 1998).

An important factor in the different structures of termite gut microbial communities in the anterior mid and hindgut is ecological niche heterogeneity (Hong et al., 2010). The biochemical environment of the termite gut has been studied by researchers in the field. The hindgut, which is full of accumulated hydrogen, is anaerobic (Tholen and Brune, 2000; Pester and Brune, 2007), which is the result of hydrogen molecules produced by dense colonies of Spirochaetes and Fibrobacteria (Brune and Friedrich, 2000; Li et al., 2012). The hypoxic abdominal environment in live termites may affect the ability of *Metarhizium* conidia to germinate (Brune et al., 1995). In addition, the digestive tract is long enough to exert a negative influence on the germination of spores (Earl et al., 2015; Hegde et al., 2019). Gut microorganisms can effectively inhibit the colonization and growth of foreign pathogens in the gut (Ivanov and Honda, 2012), in addition to passively creating an environment that is already unsuitable for *Metarhizium*. As a result, it is less necessary for the gut microbiota to actively respond to *Metarhizium* infestation than may be expected.

Gut microbe diversity decreased at 12 h post-infection, then re-stabilized over the next two time points. At 24 h, the diversity level was similar to the control, but the microbial composition of the gut was much closer to the 12 h time point. At 48 and 72 h, the microbial composition was much closer to the control, but still somewhat different. Based on our calculation of UniFrac distances, we found a significant difference in gut flora composition at 72 h post-infection and before infection. The increases and decreases in flora diversity did not affect the status of the dominant phylum Bacteroidetes, and it was mainly the intermediate status phyla that were competing for dominance. This seems to imply that some gut bacteria are not easily killed by *Metarhizium* and that these less easily killed bacteria occupy the space of bacteria more affected by *Metarhizium* infestation. In mosquitoes, previous research has found more pronounced fluctuations in gut microbiota colony composition than in termites (Scharf et al., 2017; Wei et al., 2017), and that is because termites also exhibit social immunity. For social insects, behaviors such as epidermal cleaning through inter-nestmate grooming may lead to a decrease in the rate of mortality from infection (Davis et al., 2018).

We have found that diversity of flora decreased at 12 h post-infection (Figure 2). Compared with the control group, Proteobacteria, Actinobacteria, Fusobacteria, and Acidobacteria showed a significant increase in abundance at the 12 h node, while Firmicutes and Gracilibacteria showed a significant decrease in abundance at the 12 h node. The significant changes in abundance of these phyla at the 12 h node can explain the decrease in diversity of flora, and it can be reasonably inferred that some bacteria of these phyla have developed an adaptive stress response to infection. Following Chew et al.’s (2018) work demonstrating the importance of complex interactions between different gut flora for the degradation of lignocellulose, we visualized the interactions of the gut flora, which also changed significantly during the infection of *Metarhizium*, which was echoed by changes in overall function. Because the micro-environment of the hindgut is dominated by endosymbionts, changes in interactions between bacterial phyla also reflect changes in increase and decrease of function genes. The functions of some of these phyla have been studied. Spirochetes are capable of a variety of metabolic processes, including acetate production, nitrogen fixation, and degradation of lignin phenolics (Manjula et al., 2016; Peterson and Scharf, 2016a). Its high motility allows it to colonize the hindgut environment (Brune, 2014). Gut flora disorders in the hindgut have been observed after antibiotic treatment. In response to gut disorders, spirochetes increase their capacity to process cellulose 1.6-fold (Peterson et al., 2015), but their own dominance due to antimicrobial treatment is replaced by other antimicrobial-tolerant phyla with similar metabolic capacity, a situation often thought to provide enhanced resilience of the microbial community in the face of stressful situations (Peterson et al., 2015). Its increased abundance as a hindgut representative phylum at 12 h in Figure 3B implies rescue because of the down-regulation of hindgut metabolic functions, particularly cellulolytic functions, associated with the infection of *Metarhizium*. In our study we found that the apparent upregulation of the abundance of Spirochaetes at 12 h post-infection could be the initial means by which the microbiota copes with pathogenic fungi. Based on their resilience in the face of adverse conditions, it is possible that spirochetes adapt to *Metarhizium* when faced with the presence of *Metarhizium* in the gut (Brune, 2014). Elusimicrobia, Bacteroidetes, Proteobacteria, and Actinobacteria can ferment glucose, synthesize amino acids, produce ecological factors, fix nitrogen, and recycle nitrogenous waste. Methane production in the gut of termites can be attributed to archaeobacteria (Peterson and Scharf, 2016a). Well-grown Actinobacteria were found in the termite nest environment, and its secondary metabolites have peroxidase activity (Godden et al., 1992; Kurtböke and French, 2010) and can serve as an exogenous source of resistance against *Metarhizium* (Chouvenc et al., 2013). Moreover, the phylum Actinobacteria is also the phylum in which the nest flora and hindgut overlap; whether this implies that the phylum Actinobacteria also plays a role in the resistance of the hindgut to *Metarhizium* is unclear. The antimicrobial performance of the Actinobacteria in our study was not reflected in its abundance. Possible reasons for this are that the gut environment limits the production of secondary metabolites by actinomycetes or that actinomycetes require a period of response time in response to *Metarhizium* and the production of secondary metabolites takes some time to be effective. Hosts are *usually associated with a specific group of microorganisms: 78.6% of the core microbiota of higher* termites are distributed in the phyla Bacteroides and Firmicutes, and we also found alterations in the abundance of the core microorganism Firmicutes (Otani et al., 2014). Bacteroidetes and Firmicutes (Figure 3E) are associated with fungal cell wall degradation, and it has been proposed that they are able to adapt bacteria to the gut environment through DNA transfer between themselves and other bacteria (Ning et al., 2013).

Among the functional changes in the overall abundances of gut flora, we also found a trend for most functional genes to be decreased after 12–24 h of *Metarhizium* infection, while recovering to or even exceeding the functional level of the uninfected control at 72 h when the infected termite population was almost dead. Among them, protein digestion and absorption are related to protein digestion by the gut mucosa. Upregulation of this pathway implies an increased protein demand by the host, possibly due to proteases secreted by pathogenic fungi that break down proteins in otherwise healthy worms or antimicrobial peptides secreted in response to stress. The downregulation of the Nitrogen metabolism and Methane metabolism pathways, both of which are related to nitrogen metabolism, suggests that the reduction of methanogenic bacteria in the hindgut of termites alters the biochemical environment of the hindgut. Propanoate metabolism is involved in the synthesis of insect pheromones (Madec et al., 2006), and the downregulation of this pathway implies that social contact between infected individuals is altered and that termites can prevent the spread of pathogens at its source by reducing communication with their nestmates.

The involvement of microbes in methane metabolism, aromatic hydrocarbon degradation, nitrogen metabolism, and amino acid metabolism has also been documented to indicate the involvement of hindgut microbiota in the formation of the hindgut environment (Hongoh et al., 2008a,b). The gut of termites is rich in bacterial transporters, of which ABC transporters have been found to play an important role in other insect–microbe relationships, especially in cases involving metabolic partitioning (Wu et al., 2006; Snyder et al., 2010; Oakeson et al., 2014), supporting the idea that there are many types of microbe-produced compounds utilized to protect the gut environment in the presence of pathogenic fungi. These genes encode enzymes, such as DNA Pol I, responsible for DNA repair and have been shown to respond to oxidative stress (Imlay, 2013). It has been found that termite hosts are closely associated with a specific group of microorganisms, which are called core microbiota. Most of the core taxa of cultivated termites are distributed in the Bacteroidetes and Firmicutes, and the need for a link between the composition and function of the gut flora has been described (Otani et al., 2014). The changes in diversity and composition that we observed responded functionally to the reduction of relevant pathways at 12 h post-infection, indicating the beginning of changes in the gut environment. We also identified two pathways, Protein export and Ribosome, that showed high redundancy in the pre-infection period. Upregulation of these pathways results in some genetic information being processed and translated, expressing proteins and actively translocating them out of the cell, suggesting that the gut flora in this regard may be a means of resistance to pathogens.

After examining the survival and gut flora of termites following pathogen infection, we investigated changes in gut flora from various aspects. We studied the composition, diversity, changes in phylum abundance, phylum symbiotic correlations, and predicted potential functions of gut microbes before and after *M. robertsii* infestation. We affirm that the gut flora were affected in the post-infection period and then recovered and even surpassed pre-infection flora levels and suggest that flora interactions also contribute to the functional down-regulation of most of the energy metabolic pathways that were present before the infection. Gut flora recovered most of their functional levels despite the continued attack of *M. robertsii* and its entry into the gut, continuing to provide more energy for the host to resist the pathogen.

## Data Availability Statement

The datasets presented in this study can be found in online repositories. The names of the repository/repositories and accession number(s) can be found below: NCBI; SUB8464664 and SUB8429336.

## Author Contributions

X-GZ, M-WL, and QW: conceptualization and supervision. C-YW and Y-XZ: data curation and visualization. Y-XZ: formal analysis, methodology, and software. X-GZ and QW: funding acquisition. M-WL and QW: project administration. QW: resources. C-YW, JM, and Y-XZ: writing – original draft. AM, X-GZ, and QW: writing – review and editing. All authors contributed to the article and approved the submitted version.

## Conflict of Interest

The authors declare that the research was conducted in the absence of any commercial or financial relationships that could be construed as a potential conflict of interest.

## Publisher’s Note

All claims expressed in this article are solely those of the authors and do not necessarily represent those of their affiliated organizations, or those of the publisher, the editors and the reviewers. Any product that may be evaluated in this article, or claim that may be made by its manufacturer, is not guaranteed or endorsed by the publisher.

## Footnotes

1
https://cran.r-project.org

2
http://www.genome.jp/kegg/
