# Supplementary material for: Microbial Response to Fungal Infection in a Fungus-Growing Termite, Odontotermes formosanus (Shiraki)
Source: Front Microbiol. 2021 Nov 22;12:723508. doi: 10.3389/fmicb.2021.723508 (PMC8645866; doi:10.3389/fmicb.2021.723508)
Supplement: Supplementary file 1 [file Data_Sheet_1.docx]

Supplementary Material

# Supplementary Figures and Tables

## Supplementary Figures

**Supplementary Figure 1.** Beta diversity baesd on unweighted Unifrac distances


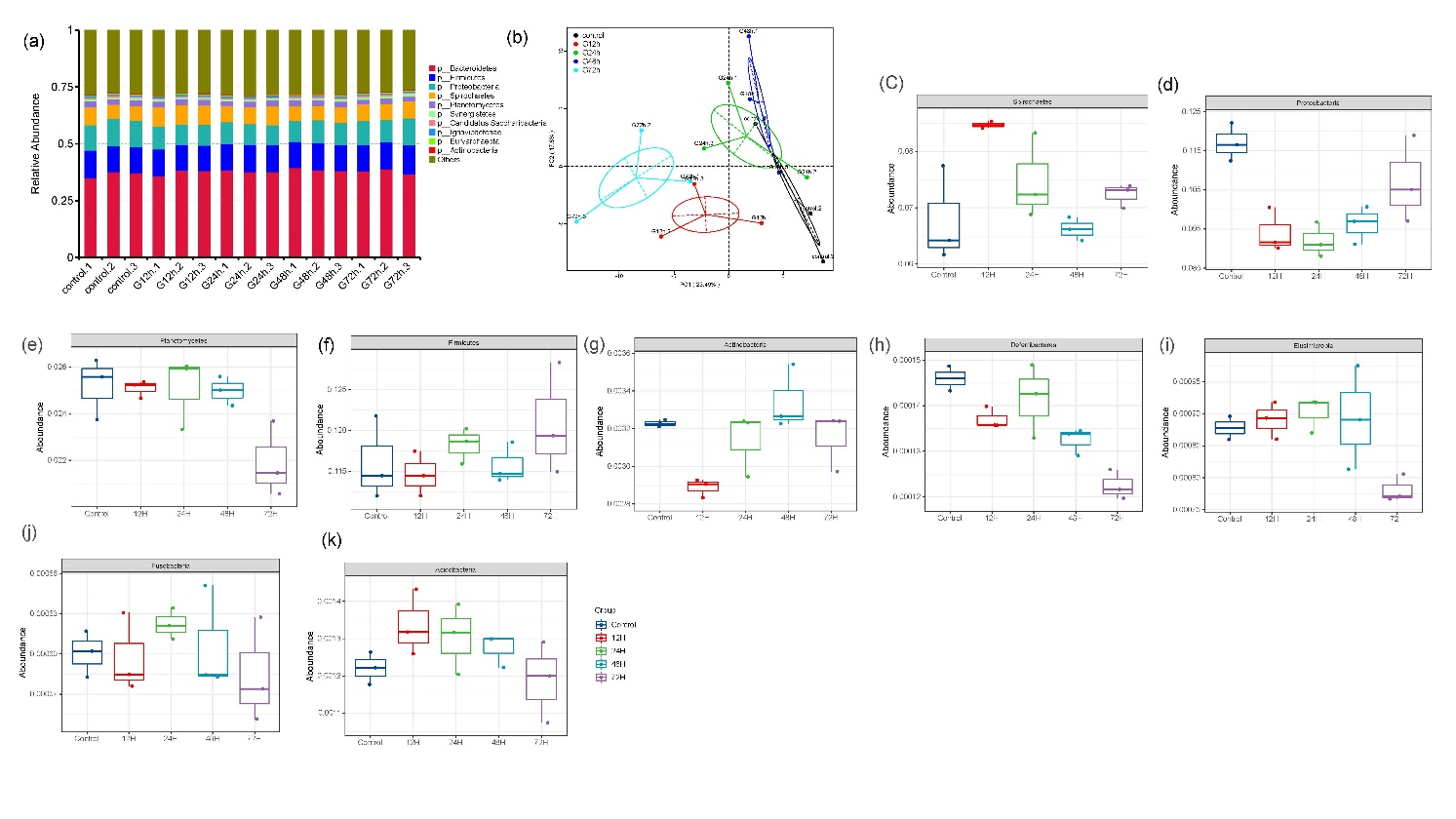


**Supplementary Figure 2.** Changes in the composition of *O. formosanus* gut microbiome following *M. robertsii* infection based on gut metagenome data. (a) Relative abundances of gut microbes at the phylum level. There are five clusters, each including three independent repetitions. Each bar graph demonstrates the microbial community composition of one independent sample in the corresponding group. (b)Principal coordinate analysis of gut microbial community composition was based on Unweighted Unifrac distance. Three replications of the same treatment group were represented by dots of the same color. There were four treatment groups and one control group. (c-k) Relative abundances over time of the significantly different phyla.

## Supplementary Tables

Supplementary Table 1. Characteristics of the shotgun metagenomic sequencing data

| Sample | InsertSize(bp) | SeqStrategy | RawData | RawReads | Low_Q | N_num | Adapter | Duplication | Poly | CleanData | Clean_Q20 | Clean_Q30 | Clean_GC(%) | Effective(%) |
| --- | --- | --- | --- | --- | --- | --- | --- | --- | --- | --- | --- | --- | --- | --- |
| control.1 | 350 | (150:150) | 8,064.46 | 53,763,060 | 0 | 0.06 | 14.62 | 0 | 0 | 8,046.27 | 96.68 | 91.34 | 51.24 | 99.774 |
| control.2 | 350 | (150:150) | 7,200.30 | 48,002,022 | 0 | 0.11 | 7.34 | 0 | 0 | 7,191.13 | 96.97 | 91.93 | 52.68 | 99.873 |
| control.3 | 350 | (150:150) | 8,385.78 | 55,905,232 | 0 | 0.07 | 9.81 | 0 | 0 | 8,373.58 | 97.28 | 92.52 | 52.86 | 99.854 |
| 12h.1 | 350 | (150:150) | 6,981.83 | 46,545,504 | 0 | 0.03 | 3.64 | 0 | 0 | 6,976.73 | 97.02 | 92.08 | 50.73 | 99.927 |
| 12h.2 | 350 | (150:150) | 7,110.25 | 47,401,678 | 0 | 0.07 | 15.05 | 0 | 0 | 7,092.10 | 97.18 | 92.34 | 51.86 | 99.745 |
| 12h.3 | 350 | (150:150) | 8,100.20 | 54,001,310 | 0 | 0.07 | 10.54 | 0 | 0 | 8,086.97 | 97.1 | 92.21 | 51.81 | 99.837 |
| 24h.1 | 350 | (150:150) | 7,931.85 | 52,879,010 | 0 | 0.11 | 4.73 | 0 | 0 | 7,925.16 | 96.91 | 91.86 | 50.33 | 99.916 |
| 24h.2 | 350 | (150:150) | 8,122.69 | 54,151,292 | 0 | 0.07 | 3.48 | 0 | 0 | 8,117.88 | 97.26 | 92.5 | 51.88 | 99.941 |
| 24h.3 | 350 | (150:150) | 7,394.41 | 49,296,068 | 0 | 0.09 | 5.18 | 0 | 0 | 7,387.47 | 96.48 | 90.84 | 50.92 | 99.906 |
| 48h.1 | 350 | (150:150) | 7,051.43 | 47,009,530 | 0 | 0.08 | 3.45 | 0 | 0 | 7,046.99 | 97.17 | 92.33 | 51.44 | 99.937 |
| 48h.2 | 350 | (150:150) | 6,502.08 | 43,347,196 | 0 | 0.09 | 6.69 | 0 | 0 | 6,493.35 | 96.93 | 91.9 | 51.19 | 99.866 |
| 48h.3 | 350 | (150:150) | 6,719.98 | 44,799,890 | 0 | 0.09 | 5.53 | 0 | 0 | 6,712.91 | 97.03 | 92.08 | 51.91 | 99.895 |
| 72h.1 | 350 | (150:150) | 7,378.07 | 49,187,110 | 0 | 0.06 | 8.02 | 0 | 0 | 7,368.08 | 97.06 | 92.06 | 52.05 | 99.865 |
| 72h.2 | 350 | (150:150) | 7,121.05 | 47,473,686 | 0 | 0.06 | 6.37 | 0 | 0 | 7,112.96 | 97.24 | 92.48 | 52.15 | 99.886 |
| 72h.3 | 350 | (150:150) | 7,399.88 | 49,332,520 | 0 | 0.06 | 9.86 | 0 | 0 | 7,388.46 | 97.03 | 91.99 | 52.66 | 99.846 |

Supplementary Table 2. Additional analyses to test for biological consistency and effect correlation using Pearson's product-moment correlation method.

| Taxon1 | Taxon2 | Correlation | P.value | Statistic | Groups |
| --- | --- | --- | --- | --- | --- |
| Acidobacteria | Unassigned | 0.8382 | 0.0371 | 3.074196 | 0h vs 12h |
| Actinobacteria | Elusimicrobia | 0.8161 | 0.0476 | 2.824703 | 0h vs 12h |
| Bacteroidetes | Elusimicrobia | -0.8587 | 0.0285 | -3.35155 | 0h vs 12h |
| Bacteroidetes | Planctomycetes | -0.9361 | 0.006 | -5.32229 | 0h vs 12h |
| Elusimicrobia | Planctomycetes | 0.921 | 0.0091 | 4.727941 | 0h vs 12h |
| Euryarchaeota | Tenericutes | -0.9072 | 0.0125 | -4.31372 | 0h vs 12h |
| Fusobacteria | Gracilibacteria | 0.9008 | 0.0143 | 4.149208 | 0h vs 12h |
| Fusobacteria | Proteobacteria | 0.8234 | 0.044 | 2.901828 | 0h vs 12h |
| Fusobacteria | unidentified_Bacteria | 0.9106 | 0.0116 | 4.405964 | 0h vs 12h |
| Proteobacteria | unidentified_Bacteria | 0.8201 | 0.0456 | 2.866384 | 0h vs 12h |
| Spirochaetes | unidentified_Bacteria | -0.8616 | 0.0274 | -3.39556 | 0h vs 12h |
| Acidobacteria | Euryarchaeota | 0.8622 | 0.0272 | 3.404148 | 0h vs 24h |
| Acidobacteria | Tenericutes | -0.9436 | 0.0047 | -5.69951 | 0h vs 24h |
| Actinobacteria | Gracilibacteria | 0.8252 | 0.0432 | 2.922047 | 0h vs 24h |
| Bacteroidetes | Planctomycetes | -0.8802 | 0.0207 | -3.70915 | 0h vs 24h |
| Bacteroidetes | Synergistetes | -0.8177 | 0.0468 | -2.84079 | 0h vs 24h |
| Deferribacteres | Gracilibacteria | 0.8935 | 0.0164 | 3.978673 | 0h vs 24h |
| Elusimicrobia | Planctomycetes | 0.9208 | 0.0092 | 4.720709 | 0h vs 24h |
| Euryarchaeota | Tenericutes | -0.8864 | 0.0186 | -3.83015 | 0h vs 24h |
| Gracilibacteria | Synergistetes | 0.8549 | 0.0301 | 3.295108 | 0h vs 24h |
| Bacteroidetes | Synergistetes | -0.895 | 0.016 | -4.01331 | 0h vs 48h |
| Elusimicrobia | Planctomycetes | 0.8783 | 0.0213 | 3.673933 | 0h vs 48h |
| Elusimicrobia | Synergistetes | 0.8755 | 0.0223 | 3.623954 | 0h vs 48h |
| Euryarchaeota | Gracilibacteria | 0.8689 | 0.0247 | 3.510526 | 0h vs 48h |
| Euryarchaeota | Tenericutes | -0.8884 | 0.018 | -3.86981 | 0h vs 48h |
| Euryarchaeota | Unassigned | 0.9432 | 0.0048 | 5.675995 | 0h vs 48h |
| Euryarchaeota | unidentified_Bacteria | 0.8498 | 0.0322 | 3.223821 | 0h vs 48h |
| Firmicutes | Spirochaetes | -0.9075 | 0.0124 | -4.32158 | 0h vs 48h |
| Gracilibacteria | unidentified_Bacteria | 0.8206 | 0.0454 | 2.871682 | 0h vs 48h |
| Planctomycetes | Synergistetes | 0.8579 | 0.0288 | 3.339985 | 0h vs 48h |
| Tenericutes | Unassigned | -0.9541 | 0.0031 | -6.37169 | 0h vs 48h |
| Unassigned | unidentified_Bacteria | 0.8839 | 0.0194 | 3.780099 | 0h vs 48h |
| Actinobacteria | Elusimicrobia | 0.9271 | 0.0078 | 4.945639 | 0h vs 72h |
| Actinobacteria | Planctomycetes | 0.8194 | 0.046 | 2.858595 | 0h vs 72h |
| Actinobacteria | Synergistetes | 0.8456 | 0.0339 | 3.168457 | 0h vs 72h |
| Bacteroidetes | Euryarchaeota | -0.9399 | 0.0053 | -5.50561 | 0h vs 72h |
| Bacteroidetes | Synergistetes | -0.9041 | 0.0134 | -4.23106 | 0h vs 72h |
| Deferribacteres | Fusobacteria | 0.8389 | 0.0368 | 3.082533 | 0h vs 72h |
| Deferribacteres | Spirochaetes | -0.8299 | 0.0409 | -2.97543 | 0h vs 72h |
| Deferribacteres | Tenericutes | 0.8922 | 0.0168 | 3.950144 | 0h vs 72h |
| Elusimicrobia | Planctomycetes | 0.9474 | 0.0041 | 5.920742 | 0h vs 72h |
| Euryarchaeota | Tenericutes | -0.8342 | 0.0389 | -3.02572 | 0h vs 72h |
| Euryarchaeota | unidentified_Bacteria | 0.9063 | 0.0127 | 4.289677 | 0h vs 72h |
| Tenericutes | unidentified_Bacteria | -0.9603 | 0.0023 | -6.88519 | 0h vs 72h |
